# Retroperitoneal Donor Nephrectomy: An Experience of 490 Patients in Mexico

**DOI:** 10.7759/cureus.97345

**Published:** 2025-11-20

**Authors:** Roberto Bautista Olayo, Ramón Espinoza Pérez, Héctor Cedillo Galindo, Jorge David Cancino López, Juan Carlos H Hernández-Rivera

**Affiliations:** 1 Renal Transplant Unit, Hospital de Especialidades, Centro Médico Nacional Siglo XXI, Mexico City, MEX; 2 Medical Research Unit in Nephrological Diseases, Hospital de Especialidades, Centro Médico Nacional Siglo XXI, Mexico City, MEX

**Keywords:** kidney donor, live donor kidneys, lumboscopic, nephrectomy, retroperitoneal

## Abstract

Background: Laparoscopic nephrectomy is the standard for kidney donors, offering the advantages of minimally invasive surgery without increasing risks to the patient or the graft. There are several transperitoneal and retroperitoneal approaches, but there is no consensus on the optimal one.

Objective: To present our centre’s experience with retroperitoneal lumboscopic nephrectomy and to report the outcomes achieved in terms of safety, donor morbidity, and graft performance.

Methods: A retrospective observational cohort analysis of patients undergoing retroperitoneal lumboscopic nephrectomy between October 2016 and December 2023 was performed. Descriptive statistical analysis was performed with median and interquartile ranges, as well as frequencies and percentages as appropriate.

Results: Between October 2016 and December 2023, we operated on 490 patients, of whom 203 were men. Left nephrectomy was performed in 486 of the surgeries. There was no mortality associated with the procedure, and morbidity was minimal. The mean surgical time was 96 minutes with a standard deviation (SD) of ± 13 minutes. Three patients (0.6%) required reoperation: two for residual hematoma and one for a chylous collection. Four patients (0.8%) required conversion to open surgery. All procured kidneys were transplanted, and the average warm ischemia time was 4.5 minutes (SD ± 1.2 min). Two of the recipients of these grafts experienced delayed graft function, which subsequently resolved, and the patient now has a functioning kidney. Postoperative outcomes were favorable, and hospital stays were less than 72 hours, except for the two patients who underwent early reoperation. Patients returned to their normal daily activities in approximately three weeks.

Conclusions: Laparoscopic retroperitoneal nephrectomy proved to be a safe and effective surgical technique with excellent results for both the patient and the graft.

## Introduction

Kidney transplantation is the optimal renal replacement therapy (RRT) that can be offered to patients with advanced kidney disease. The improvements in survival, quality of life, and function are clear and better than keeping the patient on the waiting list with dialysis [1]. Every year, the number of transplants performed worldwide increases, and so is the access to transplantation. However, there is still a wide gap between the number of people who would need a transplant and the number of organs available for transplantation.

For this reason, several strategies have been adopted to expand the pool of organs available for transplantation. These include accepting brain-dead donors who meet extended criteria due to advanced age or comorbidities [2,3], promoting donation after circulatory death [4], and increasing the number of transplants performed using living donors, despite the associated medical and social considerations [5].

All of these strategies have been implemented to a greater or lesser extent in developing countries like ours. However, some limitations, such as the logistical issues of the healthcare system or the low donation rate (4.21 per million in 2024, in data of the Global Observatory on Donation and Transplantation), mean that living donors are the primary source of transplanted organs. In Mexico, in 2024, of the 2723 kidney transplants performed, 1749 (64.2%) were from living donors [6].

With this type of donor, there are two important aspects to consider: the medium- and long-term follow-up required to monitor the "new" kidney function, the compensation they achieve, and the attention that should be paid to the evolution and presentation of comorbidities that could increase the risk of subsequent kidney problems [7]. Women of childbearing age require close care during pregnancy due to the increased risk of hypertensive disease and other complications [8]. Another very important aspect in these patients is the nephrectomy. Considering that these are healthy patients undergoing major surgery, efforts have been made to offer the lowest possible associated morbidity. For more than 30 years, open surgery with a retroperitoneal approach was the standard, but since the 1990s, when Ratner et al. initiated laparoscopic nephrectomies for donor purposes [9], it has gradually become the modality of choice. Accumulated experience showed that it was possible to offer patients the advantages of minimally invasive surgery, such as shorter hospital stays, less pain, better cosmetic results, and fewer medium- and long-term complications, while ensuring patient safety and without impacting the function of the procured grafts. This has made the laparoscopic approach the preferred in most transplant centres worldwide [10,11].

What remains a matter of debate is which of the available approaches should be considered the one of choice. Most procedures are performed transperitoneally [11] (a route that can be performed by hand-assisted, total laparoscopic, robot-assisted, among other variations), and there are also retroperitoneal approaches with good experience, both pure and with hand-assisted technique [12]. All approaches have demonstrated effectiveness, each with its own advantages and disadvantages. The transperitoneal approach offers a wider manoeuvring space but carries a higher potential risk of intra-abdominal visceral injury, ileus, adhesions, and postoperative incisional hernia [12-14]. In contrast, the retroperitoneal approach provides direct access to the kidney and avoids manipulation of the peritoneal cavity, thereby reducing the likelihood of these complications. Some reports also cite reduced bleeding and shorter operative times as additional benefits of the retroperitoneal technique [15].

Among the limitations described with this technique, a smaller working space and a higher learning curve have been mentioned [16]. At our centre, we have opted for the total retroperitoneal approach, and the primary objective of this study is to analyse and share the results obtained in terms of safety for both the patient and the graft, and secondarily to discuss the advantages and disadvantages it offers compared to what is reported in the literature of the more commonly used transperitoneal approaches.

## Materials and methods

Design

Retrospective cohort study of kidney donors at the Kidney Transplant Unit of the Centro Medico Nacional Siglo XXI in Mexico.

Patients inclusion and exclusion criteria

All patients who were kidney donors from October 2016 to December 2023 and in whom nephrectomy was performed using the lumboscopic retroperitoneal technique were considered. All patients with surgical data, complications, and a minimum follow-up of one year were included. Patients without the necessary data or those who lost to follow-up were excluded.

Data collection

Data were obtained from the physical and electronic medical records of each donor.

Ethical considerations

Both data handling and patient confidentiality were respected in accordance with internationally established ethical considerations as well as those of the hospital itself.

Statistical analysis

Data for qualitative variables are presented as frequencies and percentages. For quantitative variables, normality was assessed using the Kolmogorov-Smirnov test, and results are reported as mean with standard deviation or median with interquartile range (25th-75th percentile), depending on the distribution. Statistical analyses were performed using Microsoft Excel and SPSS version 26 (IBM Corp., IBM SPSS Statistics for Windows, Version 26.0 (2019)).

Surgical technique description

**Figure 1:**
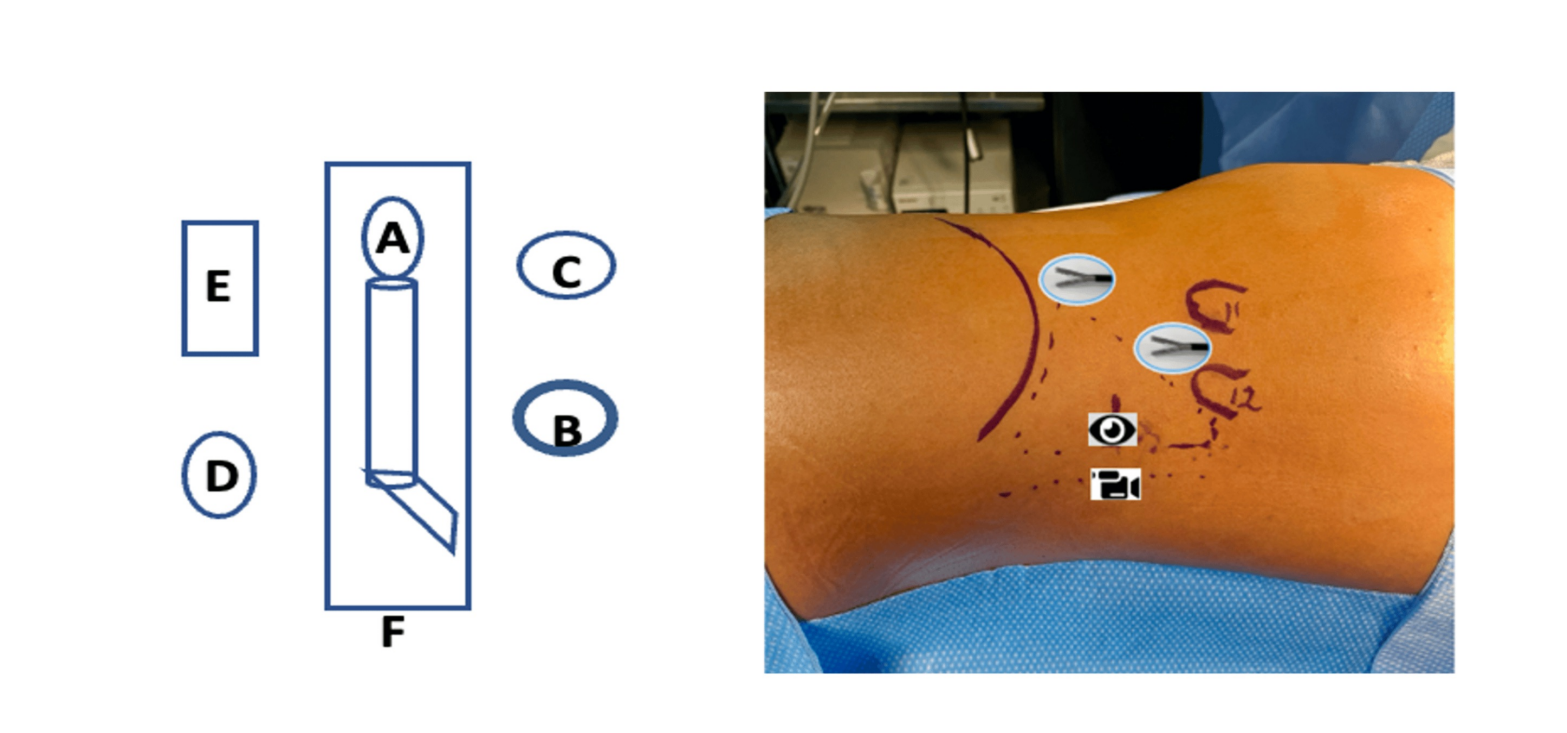
The layout of the surgical equipment and the trocar placement site for the lumboscopic retroperitoneal nephrectomy approach. A. Patient B. Surgeon C. Assistant D. Scrub nurse E. Laparoscopic equipment

With the patient positioned in the right lateral decubitus position (for left nephrectomy) and slightly flexed to improve lumbar exposure, the surgeon and assistant position themselves behind the patient and begin the procedure by approaching the upper lumbar triangle. Using the tip of the 12th rib as a reference point, an incision is made a few centimetres caudally and just above the lateral border of the erector spinae muscle. After incising the skin and subcutaneous tissue, the thoracolumbar fascia is identified by palpation and opened to access the retroperitoneal space. Digital dissection is then performed and complemented with balloon dilation to create an adequate working space. A 12-mm port is inserted at this site to serve as the visual port, and retropneumoperitoneum is established at a pressure of 10-12 mmHg. Under direct visualisation, two additional working ports are placed: a 12-mm port approximately 1 cm superior to the 11th rib along the posterior axillary line, and a 5-mm port about 0.5 cm from the anterosuperior iliac crest, following standard laparoscopic triangulation principles (Figure 1).

Under laparoscopic vision, we perform an examination that allows us to verify the dissection achieved and then proceed to locate the psoas muscle, which is the initial reference in this approach. On the inner lateral border of this muscle, the ureter can be easily identified, which is dissected distally to its intersection with the iliac vessel. We continued cephalad, completing the opening of Gerota's fascia and identifying the upper pole of the kidney. Dissecting over the gonadal vein most often identify the main trunk of the renal vein and, anterior to it, the artery. Both are freed from the surrounding lymphatic tissue, and once this is achieved, the peritoneal surface of the kidney and the fatty tissue that connects it to the adrenal gland are separated, leaving it practically free.

With the dissection complete, we performed the extraction approach, which can be performed through different routes. In most of our patients, we have opted for an incision in the left iliac fossa above the inguinal region (which allows for more direct access), but it is also possible to perform it through a Pfannenstiel incision or a mini lumbotomy. With the approach ready, we began sequential clamping of the ureter, artery, and vein, which were clipped and cut (we use polymer clips for control), after which we extracted the organ for preimplantation preparation.

## Results

Between October 2016 and December 2023, we performed a total of 490 nephrectomies with a retroperitoneal approach. Patient characteristics and some aspects of the surgery are shown in Tables 1, 2.

**Table 1:** Baseline patient and surgery characteristics (N=490). The data has been represented as N, %. n: number; kg: kilograms; m: meters.

Quantitative variables		Frequency	Percentage
Sex			
	Male	203	41.5
	Female	287	58.5
Body mass index (kg/m^2^)			
	Underweight	5	1
	Normal weight	152	31
	Overweight	270	55
	Obesity	63	13
Previous abdominal or pelvic surgery		112	22.8
Nephrectomy			
	Left	486	99
	Right	4	1
Artery			
	Single	412	84
	Multiple	78	16

**Table 2:** Baseline patient and surgery characteristics (N=490). * Expressed as Mean ± SD, as well as the minimum-maximum range.

Quantitative variables *			
Age (years)		38 ± 10.9	18-68
Body mass index (kg/m2)		26.2 ± 3.0	16-9-34.8
Surgical time (minutes)		96 ± 13	70-180
Bleeding (milliliters)		103.7 ± 13.9	80-210
Warm ischemia (minutes)		4.5 ± 1.2	3-15

It is noteworthy that these were almost all left nephrectomies. There was no procedure-related mortality, and in four cases (0.81%), a conversion to open surgery was required: two due to bleeding and two due to lack of progression of the dissection. Among the intraoperative morbidities, only one patient experienced bleeding due to a tear in the renal artery wall during the dissection, which led to conversion and was satisfactorily controlled. Eleven patients (2.2%) experienced incidental opening of the peritoneum (less than one centimeter), which did not lead to conversion. The outcome was satisfactory. Three patients (0.6%) required reoperation: two due to the presence of a hematoma requiring transfusion and examination, which was diagnosed and treated within the first 48 hours of postoperative progression. Another patient was readmitted around two weeks postoperatively after a chylous collection was detected that required drainage in the operating room (Clavien-Dindo IIIb) [17]. The patient's progress after surgery was favorable, and the patient was discharged due to improvement three days later. There were no cases of intra-abdominal visceral injury or ileus. A liquid diet was prescribed four to six hours after the procedure, with good tolerance. Pain recorded during the first 24 hours averaged 3 on a scale of 0-10. Hospital stay was less than 72 hours in almost all patients, except for the two who required early reoperation and who had a two-day longer stay than the rest of the patients without complications.

All the kidneys obtained were transplanted. Two of the recipients presented delayed graft function, which recovered before the first week. One of these grafts had a 15-minute warm ischemia as a possible associated factor, while the other had no incident or circumstance that could be related. Both kidneys are currently functional. In Table 3, we detail the transoperative or immediate postoperative incidents both with the patients and with the procured grafts that occurred in a total of 26 patients (5.3%).

**Table 3:** Perioperative and early postoperative incidents. The data has been represented as N, %. Cm: centimeters

Variables	Frequency	Percentage of patients
Patients		
Intraoperative bleeding	1	0.2
Hematoma	2	0.4
Chylous collection	1	0.2
Peritoneal opening	11	2.2
Intra-abdominal viscus injury	0	0
Ileus	0	0
Postincisional hernia	0	0
Grafts		
Superficial hematoma less than 15% of the parenchyma	17	3.4
Capsular detachment of any magnitude	5	1
Incidental polar artery ligation	3	0.6
Parenchymal laceration (less than 1 cm and 0.5 cm deep)	1	0.2

At the time of this report, and with a significant number of patients with between one and five years of post-nephrectomy follow-up, we have not had any cases of post-incisional hernia, nor have any patients been readmitted for ileus, occlusion due to adhesions, or any other abdominal complication. Patients returned to normal activities approximately 28 days after the procedure, and were able to resume higher-intensity exercise after the second month.

## Discussion

The laparoscopic approach to donor nephrectomy has become the standard. Its development and current status allow it to offer the donor the advantages of minimally invasive surgery without increasing the risk to the grafts obtained. More than 90% of kidneys transplanted in living donor procedures are procured through these routes, both transperitoneal and retroperitoneal, in their different modalities [18,19]. All techniques have demonstrated safety and good results in terms of associated morbidity, and the choice of one or the other access route depends more on local factors of the transplant program and the profile of the surgeons who perform them.

The Centro Médico Nacional Siglo XXI of the Mexican Social Security Institute was the hospital where the first kidney transplant in our country was performed in 1963. By 2023, we will have reached more than 200 kidney transplants annually. Historically, the majority have been from living donors, and we perform nearly 100 donor nephrectomies per year. Since 2016, we began offering our patients retroperitoneal lumboscopic nephrectomy, and during these first eight years, it has established itself as our procedure of choice, achieving zero mortality and low morbidity for both patients and the procured kidneys.

Local factors and the surgeon's profile influence the laparoscopic approach chosen for donor nephrectomy. In Mexico, as elsewhere, at the beginning of kidney transplant practice, surgical teams included urologists for both nephrectomy and ureteral implantation in the recipient. Many still maintain this practice today. However, as transplant programs have diversified (including, for example, liver or pancreas transplants by the same group) and with the development of academic programs for the specific training of "transplant surgeons" who perform both nephrectomy and transplantation, the profile that has predominated in teams has been that of the general surgeon rather than the urologist or vascular transplant surgeon. Since this profile is now more involved, and with prior training as an abdominal general surgeon, it is not surprising to observe that transperitoneal approaches predominate in their different modalities, since it is the route that the surgeon knows best and in which he has the greatest experience. This is very valid since, as we have mentioned, both the transperitoneal and retroperitoneal routes are safe and the results for patients and grafts are very similar.

The groups that have opted for the retroperitoneal approach have done so because it can avoid most of the complications reported with transperitoneal approaches such as ileus, hernia or potential risks of intra-abdominal visceral injury or those theoretical such as the formation of adhesions after surgery [15,20]. In the 490 patients who make up this series we did not have any of these complications, the diet starting with liquids could be established from the first hours after surgery and it is also worth noting that more than 20% of the patients we operated on had a history of previous abdominal or pelvic surgery, which in addition to potentially making the transperitoneal approach more difficult would increase the risk of postoperative adhesions by adding another procedure through a previously manipulated route. Our surgical and warm ischemia times using the retroperitoneal approach were acceptable and comparable with any of those published with both retroperitoneal and transperitoneal approaches.

Complications with the grafts were within a minimal range and none of them represented the possibility of loss. Our results corroborate the benefits described of the retroperitoneal approach, mainly decreasing the risk of ileus and the possibility of injury to intra-abdominal viscera, hernia or the potential formation of postoperative intra-abdominal adhesions. Other studies have also found the benefit of achieving less surgical time and warm ischemia by this route [18]. Despite these advantages, the retroperitoneal route is still less used than the transperitoneal route. Initial studies suggested that the working space achieved in the retroperitoneum was smaller compared to the transperitoneal route and that could make the procedure more difficult. Although the space achieved is indeed smaller, it is usually sufficient. In none of the patients we operated on, regardless of height and weight, was space limiting. Perhaps this assertion of space was influenced by reports with a retroperitoneal approach but with a hand-assisted technique [20].

In the technique that we have used and described in this work, the entire dissection is performed in a “total” retroperitoneal laparoscopic manner and the hand only enters the surgical field at the time of kidney extraction after clamping and cutting. With this, we have not had problems related to the workspace achieved, and almost all procedures have been completed without the need for conversion, even as we mentioned in those patients with a body mass index greater than 30, of which we operated on 63 patients (13%). Having the incision for extraction prior to clamping also shortens the warm ischemia times, which in our series was around four minutes. We are in the process of conducting an analysis of the learning curve associated with this approach; however, our preliminary observations align with previous reports, indicating that the technique can be adopted without significant difficulty [21].

Considering the advantages offered by the retroperitoneal approach and observing that the initially described limitations of the smaller working space and the higher learning curve can be overcome as experience is acquired, we consider that it is a technique that should be incorporated into the surgical repertoire of every transplant surgeon who, by mastering both the transperitoneal and retroperitoneal routes, would have a better chance of offering the best option according to the conditions of each patient. Mastering both techniques would place surgeons in a position to compare the approaches, initially on an individual level and subsequently in a broader context. This, in turn, would enable more robust conclusions and contribute to identifying the optimal, or “gold standard,” approach for these patients.

We consider the number of patients and the experience we have gained with the technique to be a strength of the study. It is the largest series reported in our country, and the positive results obtained will help disseminate the procedure as a very good option for donors.

The main limitation of our study is its retrospective nature, where other variables that could have been studied were not considered, and the fact that the data are from a single centre. The results would be more valid if the study included a control or comparative group with the most commonly used transperitoneal approaches, with a longer follow-up period.

## Conclusions

Laparoscopic retroperitoneal nephrectomy has been a safe and effective technique for our patients, with excellent results for both the patient and the grafts procured. It is a technique that has advantages over transperitoneal approaches and can be learned by transplant surgeons to incorporate into their surgical repertoire. However, the results presented should be considered with the caveat that it is a retrospective, single-centre and single-surgical-group study.
